# Vancomycin Utilization Evaluation at Hematology-Oncology Ward of a Teaching Hospital in Iran

**Published:** 2012

**Authors:** Afsaneh Vazin, Aziz Japoni, Sakineh Shahbazi, Mohammad Ali Davarpanah

**Affiliations:** a *Pharmaceutical Research Center, Clinical Pharmacy Department, Faculty of Pharmacy, Shiraz University of Medical Sciences, Shiraz, Iran.*; b *Professor Alborzi Clinical Microbiology Research Center, Nemazee Hospital, Shiraz University of Medical Sciences, Shiraz, Iran.*; c *Shiraz HIV/AIDS Research Center, Internal Medicine Ward, Shiraz University of Medical Sciences, Shiraz, Iran.*

**Keywords:** Vancomycin, Drug utilization research, Appropriate drug use, Serum concentration

## Abstract

The aim of the present study was to evaluate the pattern of vancomycin administration in the hematology-oncology ward of Nemazee Hospital, Shiraz, Iran. Study criteria were developed to assess the several parameters involved in vancomycin therapy. These parameters include the appropriateness of drug usage, dosage, duration of therapy, monitoring for toxicity and serum concentration monitoring. The serum concentration was measured by an automated Fluorescence Polarization Immunoassay. Clinical and preclinical parameters such as Glomerular Filtration Rate (GFR), microbial culture, antibacterial sensitivity, WBC count and fever were collected and recorded for analysis.

Sixty patients were enrolled in the study, consisting of 45 males and 15 females. The age range was 15 to 68 years. In this study, 68.63% of the vancomycin used for the patients with febrile neutropenia was compatible with the Infectious Disease Society of America (IDSA) guideline. The initial dosage of vancomycin in 68.63%, rate of infusion in 100%, and dilution of vancomycin in100%, were appropriate. Inappropriate use was more evident in the continuation of vancomycin in 50% of the patients. No appropriate dosage adjustment was done for 50% of the patients with increased serum creatinine.

Based on the results, the indication of vancomycin in febrile neutropenia was satisfactory. However, there were some required factors such as continuation of vancomycin, adjustment of dosage or interval, microbial culture, antibiotic sensitivity test before the first dose administration, measurement of serum concentration and monitoring which had to be revised in order to achieve an effective treatment.

## Introduction

Misuse of antibiotics is a problem that affects all the medical specialties on a global scale. Vancomycin is one of the key antimicrobial agents in the treatment of infections caused by Gram-positive pathogens (1). It has been manifested that treatment with vancomycin may increase the risk factor of colonization and infection with vancomycin resistant entrococci (VRE), especially among immunocompromised patients (2, 3). Therefore, appropriate use of this antibiotic is very important in preventing the transfer of VRE genes to other bacteria. This organism has become a major health issue in hospitals in North America and Europe since the first report of VRE in 1998 (4, 5). In a study previously performed in Nemazee hospital, Shiraz, southern Iran, the high prevalence of VRE was reported (6). Besides, it has been noticed that vancomycin has the highest usage in hematology-oncology ward (7). This study was only concerned with the indication of vancomycin administration, and to the best of our knowledge, there has been no drug utilization research on vancomycin in the forementioned hospital yet.

The purpose of the present study was to evaluate the indication of administration, dosage, adverse drug events (ADEs), and therapeutic drug monitoring (TDM) of vancomycin in a hematology-oncology ward of a teaching hospital in Shiraz, Iran.

## Experimental

This prospective drug utilization review (DUR) study was approved by the ethics committee of Shiraz University of Medical Sciences and was conducted in a hematology-oncology ward of Nemazee hospital in Shiraz, Iran, from May 2008 to May 2009. An informed written consent was obtained from each patient prior to the study. The inclusion criterion was receiving at least 3 successive doses of vancomycin within a one year period. Patients, for whom vancomycin was discontinued before prior to achieving a steady state were excluded. Demographic data, clinical and paraclinical data, antibiotic medication history, indication of vancomycin use, dosing regimen, rate and duration of administration, the culture report and its antibiogram were collected and recorded in special forms. All the patients were monitored until vancomycin was discontinued. Collected data were analyzed to evaluate what extent the prescription of vancomycin was in accordance with Infectious Disease Society of America (IDSA) (8); and Hospital Infection Control Practices Advisory Committee (HICPAC) guideline (9).

Initial empiric administration of vancomycin in febrile neutropenia was adapted from the IDSA guideline (8). The guideline indicates that febrile neutropenic patients with followings criteria should receive vancomycin beside other antibiotics: hemodynamic instability or other evidence of severe sepsis, pneumonia documented radiographically, positive blood cultures for Gram-positive cocci before final identification and susceptibility testing is available, clinically suspected serious intravascular catheter-related infection, skin or soft tissue infection at any site, colonization with MRSA or penicillin and cephalosporin-resistant pneumococci, severe mucositis, if fluoroquinolone prophylaxis has been given and ceftazidim is employed as empirical therapy.

The serum level concentrations of vancomycin were determined by FPIA (Fluorescence polarization immunoassay) method with the TDXflx apparatus (abbots. USA). Blood samples were taken from the patients who received vancomycin for 3 consecutive days, and just before the administration of the next dose. The samples were centrifuged for 5 min; the sera were separated and stored at -70°C. It is worth noting that the blood samples were taken as part of the specified protocol of this study, since serum drug monitoring was not a routine action of this hospital.

Data were recorded in a questionnaire designed by a clinical pharmacist and infectious specialist for vancomycin usage, administration and monitoring. One log sheet was completed for each patient. Each sheet was then reviewed by an infectious disease physician and clinical pharmacist.

*Statistical analysis*

Based on statistics analysis, Student t-test was used to compare continuous variables and results presented as Mean ± SD.

To compare appropriate and inappropriate empiric antibiotic therapies in terms of patient’s outcome and changes in clinical parameters, chi-square was used. The significance level was defined as p < 0.05. All procedures were performed using the SPSS version 15 (SPSS INC, Chicago, IL, USA).

## Results and Discussion

Out of the total 450 patients admitted between May 2008 to May 2009, 60 (13.3%) met the mentioned criteria and were enrolled into the study. Among these patients, two were excluded from the study as they were transferred to other wards and corresponding data were unavailable. The demographic characteristics and diagnosis are shown in Table 1.

**Table 1:** Data collected from 58 hospitalized patients with vancomycin treatment in a hematology oncology ward during one year

**Demographic data**		
Age, mean ± SD (range), year	36.58 ± 14.33 (15 - 68)	
Sex, Male/female ratio	44/14	
Weight, mean ± SD (range), Kg	68.05 ± 12.61 (45 - 95)	
**Infection n (%)**		
Febrile neutropenia	51 (87.93%)	
**Nonfebrile neutropenia**		
Pneumonia	2 (3.43%)	
Meningitis	1 (1.71%)	
Sepsis	1 (1.71%)	
Abscess	1 (1.71%)	
Pericarditis	1 (1.71%)	
**Renal function before vancomycin initiation n (%)**		
Normal	57 (98.2%)	
Moderate renal failure	1 (1.72%)	
**Infection type n (%)**		
Community acquired		33 (56.9%)
Nosocomial acquired		25 (43.1%)
**Treatment type n (%)**		
Empirical		57 (98.2%)
Microbiologically documented		1 (1.7%)
Duration of treatment with vancomycin mean ± SD(days)		14.8 ± 8.9

Data presented as the number of patients (percentage in brackets)

Renal function calculated based on Cockroft-Gault equation

The empirical antibiotic regimens were ceftazidime plus amikacin in 70.69% of patients, cefepime in 12.06%, imipenem in 6.89%, and other combinations in 10.34%.

Patients between 21-30 years of age received vancomycin more than other age groups. The most common reason for vancomycin use was febrile neutropenia (87.93%), followed by pneumonia (3.43%), septicemia (1.71%), meningitis (1.71%), cellulites (1.71%), abscesses (1.71%), and pericarditis (1.71%). Vancomycin was prescribed for empirical treatment in 98.2% of the cases (Table 1). Initial empiric vancomycin was considered appropriate based on IDSA guideline in 68.63% febrile neutropenic patients (Table 2). Based on the HICPAC guideline, vancomycin was considered appropriate in 80% of non-febrile neutropenic patients. After 10.018 ± 6.794 days of antimicrobial therapy, vancomycin was added to the regimen. The first dose of vancomycin was appropriate in 94.8% of the patients and dosing interval was appropriate in 96.5% of patients based on calculating clearance of creatinin (Table 2). In 35% of patients, there was a rise in serum creatinin greater than 0.5 mg/dL. No appropriate dosage adjustment was done for 50% of patients who had increased serum creatinin. The mean duration of treatment was 14.8 ± 8.9 days which was inappropriate for 50% of the occasions (Table 2).

**Table 2:** Vancomycin use, evaluation of 58 patients during one year in a hematology oncology ward.

**Appropriate vancomycin initiation,continuation**	
In febrile neutropenia1 n (%)	42 from 51 (68.63%)
In nonfebrile neutropenia 1 n (%)	5 from 7 (71.43%)
Length of therapy 1 n (%)	29 (50%)
**Other appropriate vancomycin utilization**	
Vancomycin dilution n (%)	58 (100%)
Initial dosage n (%)	55 (94.8%)
Dosing interval n (%)	56 (96.5%)
Maintenance dosage n (%)	29 (50%)
Rate of infusion n (%)	58 (100%)
Correcting dosage based on creatinin clearance 3 n (%)	10 (17.23%)
Appropriate therapeutic level 2 n (%)	25 (43.1%)

1. Compare with IDSA 2010 guideline.

2. Minimum trough serum concentration should be above 10 mg/L and in complicated nfections(bacterimia, endocarditis, osteomyelitis, meningitis and hospital acquired pneumonia). trough serum concentrations of 15-20 mg/L are recommended.

3. Renal function calculated based on Cockroft-Gault equation.

Vancomycin trough serum concentration range was 15.59 ± 13.02 μg/mL (Minimum trough serum concentration should be above 10mg/L). While sub therapeutic trough level was detected in 3.6%, 53.3% had a level above the maximum therapeutic concentration (Table 2). The trough serum concentration of two patients with pneumonia was 4.60 μg/mL and 28.64 μg/mL. The trough serum concentration of one of the patient with meningitis was 8.35 μg/mL. None of the trough serum concentrations of patients with meningitis and pneumonia were in therapeutic range of 15-20 μg/mL.

No case of Redman syndrome was detected. There was no statistically significant difference between the serum concentration of patients with developed renal dysfunction and the others. Four pathogenic bacteria were isolated from the patients, including *Entroccocci, Diphtroid, E coli *and *Pseudomonas aeraginosa*.

Of the patients who received vancomycin, 81% were discharged and 19% expired. A log sheet consisting of 19 variables was completed for each patient, and variables such as route of administration (100%), rate of infusion (100%), stability condition (100%), dilution (100%), monitoring of serum creatinin (100%) were performed based on a standard guideline (Table 2) , while adjustment of dosage or interval based on calculating clearance of creatinin (50%), duration of treatment with vancomycin (50%) , microbial culture and sensitivity test before the first dose of Vancomycin (17%), repeat of culture after 72 h (93.7%), and measuring serum concentration (100%) were not compatible with the guideline.

To minimize the emergence of resistant bacteria, antibiotics need to be restricted to appropriate indication (10).

In this study, about 68.63% of vancomycin administration in febrile neutropenia patients was consistent with IDSA guideline. All of these patients received vancomycin empirically. The high rate of empirical vancomycin therapy in this study could be due to high rate of febrile neutropenia among our patients.

According to the HICPAC guideline, vancomycin was considered appropriate for 71.43% of non-febrile neutropenic patients. In a study performed in Hong Kong during an 11 week program, vancomycin was considered appropriate in 46% of courses according to CDC guideline (11). In a retrospective study performed in Singapore on 96 pediatric patients with 96 courses of vancomycin prescribed, 64.6% of courses were consistent with the HICPAC guideline (12). In other studies, the rate of inappropriateness of vancomycin orders range from 24 to 65% (13-18). Studies have shown that routine addition of vancomycin to initial empiric antibiotic regimen in febrile neutropenia has not been associated with clinical benefits (19-21). In a meta-analysis report from seven randomized controlled trials, it has been shown that addition of antibiotic to coverage Gram-positive bacteria to standard empiric therapy did not reduce mortality rate in patients with cancer and febrile neutropenia (22). Another reason to avoid routine vancomycin use in febrile neutropenia is the risk of acquiring VRE (23). Nevertheless, some Gram-positive bacterial infections may be susceptible only to vancomycin. Therefore, if these patients are not promptly treated, it could be dangerous and may even cause death. Based on IDSA guideline, inclusion of vancomycin in initial empiric therapy was considered appropriate only for high risk patients (8). Administration of vancomycin in febrile neutropenic patients of this study was not as a primary antibiotic regimen. In fact, in most of them vancomycin was added to antibiotic regimen when patients clinically deteriorate or in case of hemodynamic instability despite initial antibiotic regimen (8).

MRSA was not isolated from the cultures, and only 4 cultures out of 58 contained *entrococci, Pseudomonas aeruginosa, E coli *and *diphtroid*. The reason for low isolated bacteria from target patients could be receiving other antibiotics prior to vancomycin administration. Therefore, it is reasonable to observe that most of the cultures turn to negative due to bactericidal and static effect of the previously consumed antibiotics.

Regarding the adverse events, in this study Redman syndrome was not observed because the infusion time was appropriate in 100% of the patients. Most of the time, when a large dose of vancomycin is infused too rapidly, Redman syndrome occurs (24-26). To minimize this adverse effect, 1 g vancomycin should be administered over an infusion period of at least one hour. The infusion time should be extended for higher dosage (e.g. 2 g over 1.5-2 h)

Nephrotoxicity occurred in 35% of the patients; however, all the patients received amikacin or amphotricine B simultaneously. The nephrotoxicity potential of vancomycin monotherapy is uncommon. However, most studies suggest increasing incidence and severity of renal insufficiency emerging, when vancomycin is administered in combination with an aminoglycoside (27-29). Increase of nephrotoxicity may also occur, when conventional amphotricin B is also co-administered with vancomycin (30). The nephrotoxicity result in the present study is comparable with other reports. Some investigators recommend TDM in order to decrease the rate of nephrotoxicity, however, Darko et al. found therapeutic drug monitoring to be cost effective only in patients admitted to ICUs, those receiving another nephrotoxic drugs and possibly for oncology patients (24). Available evidence does not support the fact that monitoring serum vancomycin concentrations could help decrease the frequency of nephrotoxicity (31). Since this study was conducted on oncology patients most of whom received nephrotoxic drug simultaneously, monitoring of serum vancomycin level was justifiable. No relationship between vancomycin serum concentration and nephrotoxicity was found in the present study.

The recommended trough serum level of vancomycin is concentration above 10 μg/mL, because staph aureus strains can develop resistance with exposure to trough serum concentration of less than 10 μg/mL (31). Studies regarding vancomycin have shown that AUC/MIC ≥ 400 is necessary to achieve clinical effectiveness (32, 33). To achieve this target for a microorganism with an MIC 1 μg/mL, the trough level would have to be at least 15 μg/mL (31). In complicated infections such as meningitis, hospital acquired pneumonia, bacterimia, endocarditis and osteomyelitis, trough serum concentrations range of 15-20 μg/mL are required to improve penetration to site of infection and clinical outcome (31). 43.1% of our patients had a trough level within therapeutic range (15.59 ± 13.02 μg/mL); most of the patients had supra-therapeutic levels (53.3%). Subsequent dosage adjustment was not based on blood level monitoring, since all drug levels were measured at the end of the study and no equipment for TDM of vancomycin was present in this hospital. While based on consensus review of vancomycin, dosing of drug should be based on actual body weight and then adjusted based on trough serum concentration (31). So measuring trough serum concentration and making dose adjustment based on drug level and optimal AUC/MIC is vital to achieve more effective vancomycin therapy.

In the present study, all the patients received a fixed dose of vancomycin (1g Q12h) regardless of their actual body weights. loading dose was not considered for all the patients. However, 1.7% and 3.4% of the patients had meningitis and pneumonia, respectively. These data suggest that according to IDSA guideline, a loading dose of 25-30mg/Kg could be considered for such patients in order to achieve rapid target concentration (31). So, considering fixed dose of vancomycin for all of our patients was not compatible with the standard guideline.

In the present study the length of empiric therapy was inappropriate in 50% of the patients. It seems that inappropriate duration of vancomycin use was more common as compared with it initiation.

The results of the present study suggest that intervention need to be implemented to improve the use of vancomycin. In addition, physicians should be educated to consider some points such as, reducing their fear of de-escalation, treat patients according to results of culture and antibiogram and adjustment of dose based on trough serum level.

The major limitation of this study was the small number of patients under study, who received vancomycin.

## Conclusion

The most evident problem in vancomycin utilization in this hematology-oncology ward is that vancomycin was not stopped at proper time and continued more than it was actually needed. Another considerable problem in vancomycin usage in Iran is the ignorance of the important role of measuring vancomycin level in optimizing vancomycin use. Providing equipment and trained personnel for TDM, Training health care providers, using antibiotic stop order 72 h after the initiation of vancomycin, supervision of pharmacy and therapeutic committee on continuation of vancomycin and availability of a clinical pharmacist in hospital are recommended.
